# Circulating extracellular vesicle-enriched fraction in heart failure with reduced ejection fraction and incident cancer

**DOI:** 10.1093/ehjopen/oeag180

**Published:** 2026-10-26

**Authors:** Eva Bianconi, Antonella De Palma, Davide Perico, Silvia Bruno, Francesca Fumagalli, Enrico Nicolis, Eliana Rulli, Davide Ceresa, Ambra Costa, Chiara Vitale, Roberto Latini, Pier Luigi Mauri, Pietro Ameri

**Affiliations:** Translational Cardiovascular Unit, AOM IRCCS Ospedale Policlinico San Martino, Largo Rosanna Benzi 10, 16132 Genova, Italy; Institute for Biomedical Technologies, National Research Council (ITB-CNR), Via Fratelli Cervi 93, 20054 Segrate (MI), Italy; Institute for Biomedical Technologies, National Research Council (ITB-CNR), Via Fratelli Cervi 93, 20054 Segrate (MI), Italy; Department of Experimental Medicine, University of Genova, Via Leon Battista Alberti 2, 16132 Genova, Italy; Department of Acute Brain and Cardiovascular Injury, Istituto di Ricerche Farmacologiche Mario Negri IRCCS, Via Mario Negri 2, 20156 Milano, Italy; Department of Acute Brain and Cardiovascular Injury, Istituto di Ricerche Farmacologiche Mario Negri IRCCS, Via Mario Negri 2, 20156 Milano, Italy; Department of Oncology, Laboratory of Methodology for Clinical Research, Istituto di Ricerche Farmacologiche Mario Negri IRCCS, Via Mario Negri 2, 20156 Milano, Italy; Department of Experimental Medicine, University of Genova, Via Leon Battista Alberti 2, 16132 Genova, Italy; Translational Cardiovascular Unit, AOM IRCCS Ospedale Policlinico San Martino, Largo Rosanna Benzi 10, 16132 Genova, Italy; Department of Experimental Medicine, University of Genova, Via Leon Battista Alberti 2, 16132 Genova, Italy; Department of Acute Brain and Cardiovascular Injury, Istituto di Ricerche Farmacologiche Mario Negri IRCCS, Via Mario Negri 2, 20156 Milano, Italy; Institute for Biomedical Technologies, National Research Council (ITB-CNR), Via Fratelli Cervi 93, 20054 Segrate (MI), Italy; Translational Cardiovascular Unit, AOM IRCCS Ospedale Policlinico San Martino, Largo Rosanna Benzi 10, 16132 Genova, Italy; Department of Internal Medicine, University of Genova, Viale Benedetto XV/6, 16132 Genova, Italy

**Keywords:** Cardio-oncology, Proteomics, Mechanisms, Translational

## Abstract

**Aims:**

Previous studies found increased cancer incidence in patients with heart failure with reduced ejection fraction (HFrEF). We sought to explore the biological correlates of the association of HFrEF with cancer in humans.

**Methods and results:**

Among participants in the Gruppo Italiano per lo Studio della Sopravvivenza nell’Insufficienza Cardiaca—Heart Failure (GISSI-HF) trial with HFrEF and available baseline plasma samples, we selected those who developed solid cancer (cases) and matched them with controls without cancer by age, sex, New York Heart Association class, and follow-up duration. We isolated the plasma extracellular vesicle (EV)-enriched fraction, in which EVs were confirmed by western blot for CD9, FLOTILLIN-1, ALIX, and TSG101, and by nanoparticle tracking analysis (mean particle diameter <200 nm). The protein content of EV preparations was characterized by nanoliquid chromatography-high=resolution tandem mass spectrometry. Of the 940 identified proteins, 148 were differentially expressed between EV preparations from cases (EVs-cases) and controls (EVs-controls) and were related to biological functions implicated in cancer growth, particularly apoptosis and spliceosome/transcriptome regulation. Next, we explored the effects of EVs-cases and EVs-controls in MCF7 breast cancer cells and ran confirmatory experiments with another two cell lines, HCT116 (colon) and A549 (lung). Apoptosis, as determined by caspase 3 cleavage, was blunted by EVs-cases in MCF7, but not in HCT116 and A549 cells. Screening qPCR identified *NR3C1*, encoding the glucocorticoid receptor, as a spliceosome/transcription modulator affected by EVs-cases. Targeted qPCR confirmed that it was reduced by EVs-cases in A549 cells.

**Conclusion:**

Circulating EV-enriched fractions from patients with HFrEF and incident cancer have a distinct proteomic signature and might modulate human cancer cell biology.

## Graphical Abstract

**Graphical Abstract:**
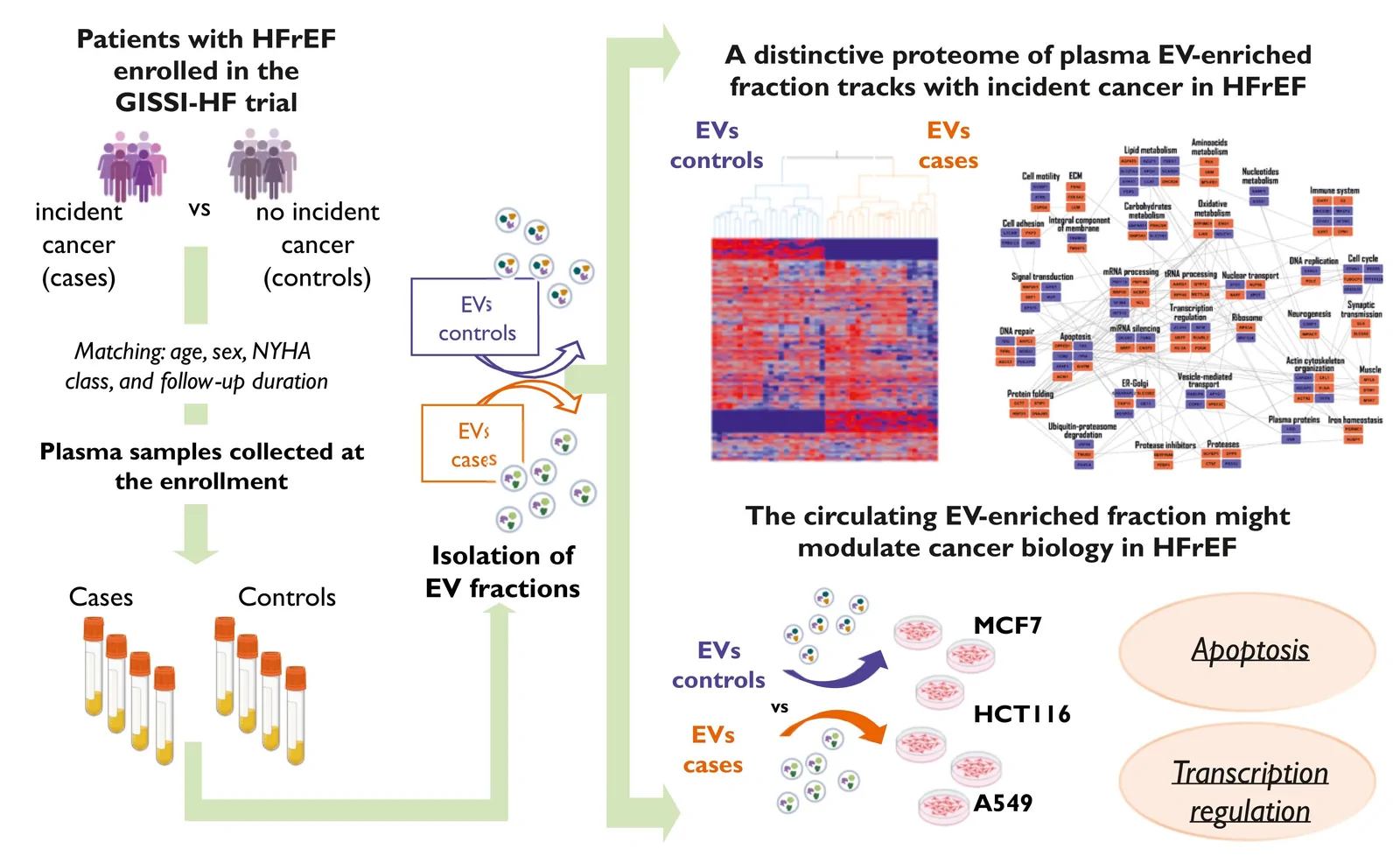
Circulating EV-enriched fractions from patients with heart failure, reduced ejection fraction and incident cancer have a distinctive proteomic signature and might exert tumour-modulating effects. Comparative analysis of the proteome of EV-enriched fractions isolated from plasma of patients with HFrEF, who were enrolled in the GISSI-HF trial and developed (cases) or did not develop (controls) cancer during the follow-up, revealed significantly different abundances of several proteins involved in cancer-related biological pathways. Preliminary experiments suggested that some of these pathways were differentially modulated by EV preparations from cases or controls in the human cancer cell lines MCF7, HCT116, and A549.

## Introduction

Several studies suggest that patients with heart failure (HF) are more likely to be diagnosed with and die from cancer, as compared with individuals without HF and after accounting for possible confounders.^1^ With the exception of prostate cancer, most malignancies have been associated with HF, including those of the breast, lung, and gastrointestinal tract.

The association of HF with cancer may be due to genetic predisposition and shared social determinants, risk factors, and pathogenic pathways.^2–4^ Moreover, enhanced tumour burden was demonstrated in mouse models of breast, lung, or colorectal cancer after inducing HF with reduced ejection fraction (HFrEF).^5^ Increased tumour growth in this setting was related to circulating factors,^6^ inhibition of tumour-controlling immune responses,^7^ and HF-induced microbial dysbiosis.^8^

More recently, it was suggested that HF sustains tumorigenesis *via* extracellular vesicles (EVs). These are lipid bilayer-enclosed, cell-derived multimolecular assemblies containing single- and double-stranded nucleic acids, proteins, lipids, and other molecules, which act as biosignals between distant tissues.^9^ In mice, EVs from the failing heart were shown to stimulate cancer growth by both inducing cancer cell proliferation and skewing tumour-infiltrating macrophages towards a tumour-permissive phenotype.^10^

However, there is limited and indirect evidence that the mechanisms by which HF favours tumorigenesis in animal models, particularly through circulating EVs, are operative in humans.

We sought to bridge this knowledge gap by exploring the biological correlates of incident cancer in a well-characterized HF patient cohort.

## Methods

The full materials and methods are available as Supplementary material.

### Plasma samples

Gruppo Italiano per lo Studio della Sopravvivenza nell’Insufficienza Cardiaca—Heart Failure (GISSI-HF) trial was a double-blind, placebo-controlled trial randomizing patients with chronic HF to n-3 polyunsaturated fatty acids and rosuvastatin.^11^ Baseline peripheral blood samples were collected at enrolment, aliquoted, and stored for future research use. Furthermore, information on incident cancer was recorded during follow-up.^12^ We selected the patients with HFrEF, who did not have any tumour at baseline but were hospitalized for or died from solid cancer during the trial (cases), and we matched them for age, sex, New York Heart Association (NYHA) class, and follow-up duration with HFrEF patients without cancer throughout the study (controls). Plasma aliquots from these patients were retrieved for EV preparation, protein extraction, and proteomic analysis.

### Extracellular vesicle–enriched fraction isolation

The EV-enriched fraction was extracted from plasma samples by means of the ExoQuick ULTRA kit (System Biosciences, Palo Alto, CA, USA). This is a polymer-based precipitation method with a post-precipitation cleanup step to reduce carryover of abundant soluble plasma proteins, such as albumin and immunoglobulins. Nevertheless, non-vesicular extracellular particles and free proteins can be co-isolated with EVs with this technique. Part of the EV-enriched fraction of every sample was used for proteomic analysis. The remaining material was pooled together to form one batch of EV preparation from cases (EVs-cases) and one batch of EV preparation from controls (EVs-controls). The concentration of EVs in both pools was measured as particles/mL by nanoparticle tracking analysis (NTA) with NanoSight NS300 (Malvern Panalytical, Malvern, UK). Moreover, the presence of the EV markers CD9 (ab236630, diluted 1:2000; Abcam, Cambridge, UK), ALIX (ab186429, diluted 1:1000; Abcam), FLOTILLIN-1 (ab133497, diluted 1:10 000; Abcam), and TSG101 (T5701, diluted 1:1000; Sigma Aldrich) was assessed by western blot.^13^ On the other hand, non-EV markers were searched in the list of identified proteins obtained by proteomic analysis.

### Protein extraction and enzymatic digestion

Protein content of the EV preparations was quantified (SPN Protein Assay, G-Biosciences, St. Louis, MO, USA), and 50 µg was treated with RapiGest SF reagent (Waters, Co, Milford, MA, USA) at 95°C for 15 min, then digested overnight at 37°C with Trypsin/Lys C Mix (Promega, Inc., Madison, WI, USA). Digestion was stopped with 0.5% trifluoroacetic acid (Sigma-Aldrich, Inc., St. Louis, MO, USA) and the peptide mixtures were desalted using Pierce C-18 spin columns (Life Technologies, Italy) and resuspended in 0.1% formic acid (Sigma-Aldrich, Inc.) in water at a concentration of 0.1 µg/µL.

### Liquid chromatography-mass spectrometry

Peptide mixtures were analysed using Eksigent nanoLC-Ultra 2D System (Eksigent, part of AB SCIEX Dublin, CA, USA) with cHiPLC-nanoflex system (Eksigent) in trap-elute mode and separated on a ChromXP C18-CL column via a 95-min gradient. Detection was performed on an Orbitrap Exploris 120 mass spectrometer (Thermo Fisher Scientific, San Josè, CA, USA) equipped with EASY-Spray ion source at 1.6 kV and 220°C (Thermo Fisher Scientific). Data-dependent acquisition was performed over an m/z range of 375–1250 at 60 000 resolution (FWHM, m/z 200). Fragmentation was performed using high-energy collisional dissociation with high-resolution MS2 scans, employing normalized collision energy and dynamic exclusion to enhance peptide identification. Full and tandem mass spectra were acquired in profile and centroid mode, respectively. Acquisition was managed viaXcalibur data system version 4.4 (Thermo Fisher Scientific) and Eksigent Control Software version 4.3 (Eksigent). Mass spectrometry (MS) data were deposited in ProteomeXchange via PRIDE^14^ and are publicly available with the accession number MSV000095415.

### Proteomic analysis

The raw data generated by nanoliquid chromatography-high-resolution tandem MS/MS were searched in Proteome Discoverer v2.5 (Thermo Fisher Scientific) using Sequest HT (UniProt database *Homo sapiens*, March 2021) and MSPepSearch (NIST HCD spectral library). Peptide-spectrum matches were validated in Proteome Discoverer v2.5 using Percolator with a target–decoy strategy, applying a false discovery rate threshold of 1%. Label-free protein quantification was performed using a pairwise ratio–based approach, which accounts for missing values in some samples. For the two conditions of interest (cases and controls), all replicates with measured values were paired and peptide abundance ratios were calculated across replicate pairs. Then, the geometric median of the abundance ratios was computed. Differentially expressed proteins (DEPs) were defined by log2 fold change (FC) ≥ │1.5│and *P*-value <0.001 (Benjamini-Hochberg). Heatmaps and principal component analysis were generated in JMP v15 (SAS Institute Inc.), and clustering used Euclidean distance with Ward’s method. Cellular component enrichment and comparison with Vesiclepedia were performed using FunRich v3.1.3 (accessed 10 June 2025).^15^ A protein–protein interaction network was created by using the STRING database,^16^ which considers only experimentally and database-defined protein-protein interactions with a score >0.4. The resulting sub-network was visualized and analysed through Cytoscape v. 3.10.1.^17^ To enhance interpretation, proteins were organized into functional modules with the support of STRING enrichment.

No formal sample size calculation was done. Instead, the numerosity of the study cohort was dictated by the number of plasma samples of at least 200 µL available from cases after the selection process described above.

### Cellular experiments

The effects of EVs-cases and EVs-controls were tested in the human MCF7 breast adenocarcinoma cell line and—in confirmatory experiments—in the human HCT116 colon carcinoma and A549 lung adenocarcinoma cell lines.

These cell lines, which represented part of the spectrum of cancers in cases, were provided by the Interlab Cell Line Collection of IRCCS Ospedale Policlinico San Martino (Genova, Italy). Cells were maintained in RPMI 1640 medium (RPMI) supplemented with 10% of foetal bovine serum (FBS), 2% L-glutamine, and 1% penicillin-streptomycin (Euroclone, Milano, Italy) at 37°C with 95% of humidity and 5% of CO_2_. Treatment with EV-enriched fractions was done for 24 h in RPMI + 1% FBS. For the experiments with MCF7, the ExoQuick ULTRA buffer, in which the EV preparations were diluted, was also added to RPMI + 1% FBS as control condition (vehicle). Apoptosis was stimulated by further 24-hour incubation with doxorubicin (Dox) 4 µM.^18^

### Cellular viability

Cellular viability was evaluated by means of a MTS-based assay (Cell Titer 96 AQueous One Solution Cell Proliferation Assay, Promega, Inc.).

### Apoptosis

The ratio between of cleaved caspase 3 and total caspase 3 was determined by western blotting with anti-cleaved caspase 3 and anti-total caspase 3 antibodies diluted 1:1000 (#9664 and #14220, respectively; Cell Signaling Technologies, CST, Danvers, MA, USA).

### Proliferation

DNA duplication was investigated with Click-iT EdU Alexa 555 HCS (Thermo Fisher Scientific), and distribution in prophase and anaphase was quantified by aurora B kinase immunostaining (ab2254, diluted 1:50; Abcam, Cambridge, UK). Images of EdU and aurora B kinase staining were acquired with Axio Imager M2 (Zeiss, Oberkochen, Germany), and positive cells were counted with Fiji-ImageJ.

### Energy metabolism and oxidative stress

Mitochondrial metabolism was studied by using an XF24 extracellular flux analyser (Seahorse Biosciences, Agilent Technologies, Santa Clara, CA) in 16-well SeaHorse XFp Cell miniplate (Seahorse Bioscience). Oxygen consumption rates were calculated based on the recorded extracellular fluxes. The amount of reactive oxygen species (ROS) was measured as median fluorescence with CytoFLEX (Beckman-Coulter, Pasadena, CA, USA) after incubation with the CellROX Deep Red Reagent (Thermo Fisher Scientific), as quantified by FlowJo 10.10 software.

### Phosphokinase screening

Phosphorylation of a panel of intracellular kinases and two related proteins with possible involvement in regulation of apoptosis was screened using Proteome Profiler Human Phospho-Kinase Array Kit (R&D systems, Minneapolis, MN, USA).

### HSP60 localization

MCF7 were seeded in eight-well chamber slides, incubated with vehicle or EV-fractions, formalin-fixed, and immunostained with an anti-HSP60 monoclonal antibody (1:50; MA3012, Thermo Fisher Scientific) and a rabbit polyclonal antibody against TOM20 (1:200; # 42406, CST). Mitochondrial reticulum and heat shock protein 60 (HSP60) fluorescence were detected on the laser scanning spectral confocal microscope TCS SP2 AOBS (Leica, Heidelberg, Germany), and co-localization of these proteins was quantified as overlapping HSP60 and TOM20 pixels with background threshold set by Costes’ method.^19^

### Gene expression analysis

Total RNA was retrieved with EuroGOLD TriFast reagent (Euroclone), purified with Quick-RNA Minipreps (Zymo Research, Irvine, CA, USA), and reverse transcribed with iScript Advanced cDNA Synthesis kit for RT-qPCR (Bio-Rad, Hercules, CA, USA). The expression of select genes with anticipated roles in regulation of transcription and spliceosome was screened in MCF7 cells with SYBR-dye-based chemistry assays; then, *AQR* and *NR3C1* normalized expressions were assessed with probes from Integrated DNA Technologies (IDT, Coralville, IA, USA; Supplementary material online, *Table S1*) and Luna Universal Probe qPCR Master Mix 2X (New England Biolabs, Ipswich, MA, USA) in MCF7, HCT116, and A549 cells. Amplifications were performed with CFX Touch (Bio-Rad), and relative gene expression was calculated.

### Statistical analysis

Data from cell experiments were compared by Student’s *t*-test, one-way ANOVA with Tuckey’s *post hoc* test, or Kruskal–Wallis test with Dunn’s *post hoc* test, depending on the distribution, by using Graph Pad PRISM 10. Statistical significance was set at *P* < 0.05.

## Results

### Proteomic profile of the extracellular vesicle–enriched fraction associated with heart failure with reduced ejection fraction and incident cancer

Among the participants in GISSI-HF with available baseline blood samples, 47 were diagnosed with or died from cancer during follow-up (cases) and 1197 did not (controls). After applying the exclusion criteria shown in Supplementary material online, *Figure S1*, 30 out of 37 cases could be matched to an equal number of controls by age, sex, NYHA class, and follow-up duration. The mean age of the selected patients was 65.9 ± 7.6 years, 87% were male, and 70% and 30% had NYHA class II and III–IV, respectively; a mean left ventricular ejection fraction was 31.4 ± 5.9% (see Supplementary material online, *Table S2*). The amount of plasma was not enough in eight patients with incident cancer. The samples from the remaining 22 cases were processed along with the samples from the 22 corresponding controls.

The isolated EV preparations expressed CD9, FLOTILLIN-1, ALIX, and TSG101 (*Figure 1A*). In addition, NTA showed that pooled EV preparations from controls and cases contained 2.35 × 10^11^ or 1.59 × 10^11^ particles/mL, respectively, and that the mean particle diameters were lower than 200 nm^13^ (*Figure 1B and C*).

**Figure 1:**
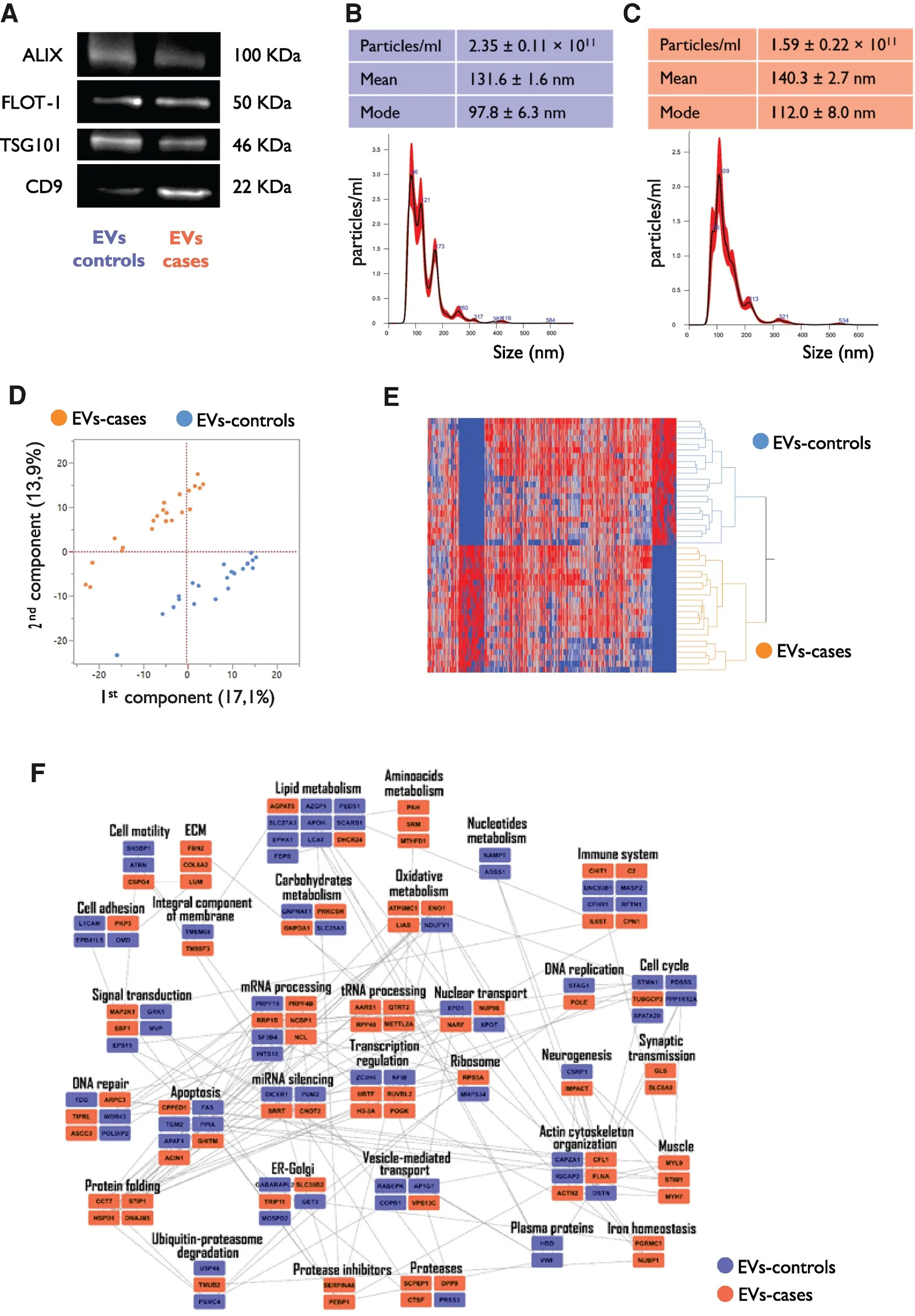
Characterization of plasma EV preparations and analysis of DEPs between EVs-controls and EVs-cases. (*A*) Expression of the EV markers ALIX, FLOTILLIN-1, TSG101, and CD9 in EVs-controls and EVs-cases, as assessed by western blot analysis. (*B–C*) NTA of EVs-controls (*B*) and EVs-cases (*C*). The concentrations and diameters (mean, modal value, and SD) are presented in the tables. (*D*) Principal component analysis of proteomic profiles across all biological replicates in EVs-cases (orange) and EVs-controls (blue). (*E*) Hierarchical clustering of the full list of proteins quantified in plasma EVs-cases (orange) and EVs-controls (blue). The dendrogram was generated based on protein abundance values across biological replicates, using Euclidean distance and Ward’s linkage method, implemented in JMP software. The heatmap colour scale reflects protein abundance levels, with blue indicating the lowest and red the highest abundance. (*F*) Protein–protein interaction network, built using the STRING database and including known and predicted interactions. DEPs were clustered into functional groups according to their molecular function, as identified by the STRING enrichment plug-in, using GO biological processes, KEGG, and Reactome pathways. The functional groups and their interrelations were visualized in Cytoscape (v3.10.1) Each protein is colour-coded in orange or blue, depending on whether it was enriched in EVs-cases or EVs-controls, respectively. DEP, differentially expressed protein; EV, extracellular vesicle.

Across all analysed samples, 940 master proteins were identified by detecting at least one unique peptide (see Supplementary material online, *Table S3*). These proteins largely overlapped with the genes in Vesiclepedia, a comprehensive EV database including 13 550 entries derived from 813 experiments (see Supplementary material online, *Figure S2A*), and the proteins identified in plasma from HFrEF patients were primarily associated with typical EV-related components, such as exosome-related compartments, cytosol, and plasma membrane, in enrichment analysis of cellular components (see Supplementary material online, *Figure S2B*). However, proteomic analysis also revealed category 3 and 4 markers of non-vesicular extracellular particles (see Supplementary material online, *Table S3*), indicating that plasma EV preparations were not pure.^13^

More than 90% of the proteins detected in EV-enriched fractions were shared between cases and controls, and the distributions of normalized protein abundances were similar between the two groups (see Supplementary material online, *Figure S3*). Nonetheless, hierarchical clustering and principal component analysis well separated cases and controls (*Figure 1D and E*). By applying a log2 FC >1.5 and adjusted *P*-value <0.001 threshold, 148 proteins were differentially expressed between EVs-cases and EVs-controls, with 73 being upregulated in the former and 75 in the latter (see Supplementary material online, *Table S4*). By using the STRING database and integrating the resulting clustering with the inputs from GO biological processes, Kyoto Encyclopedia of Genes and Genomes (KEGG), and Reactome pathways, we drew a protein–protein interaction network comprising 134 nodes and 170 edges. The network highlighted several biological functions potentially implicated in cancer growth, which were differentially represented in EVs-cases vs. EVs-controls (*Figure 1F*).

### Biological activities of the extracellular vesicle–enriched fraction associated with heart failure with reduced ejection fraction and incident cancer

Guided by the protein–protein interaction network based on the DEPs between EVs-cases and EVs-controls, we explored the effects of the EV-enriched fractions on cancer-related biological functions.

#### Effects on MCF7 cells

First, we employed the human breast cancer cell line MCF7.

Incubation of MCF7 cells for 24 h with HFrEF EV preparations at concentrations from 1 × 10^9^ to 1 × 10^4^ did not influence viability, as quantified by MTS-based assay (see Supplementary material online, *Figure S4*). Other authors evaluated the effects of HF-derived EVs at a concentration of 1 × 10^8^ EVs/mL in murine tumour cells.^10^ Since we did not observe any change in MCF7 viability across the range of tested concentrations, we consistently used a similar load of EV-enriched fractions (1 × 10^8^ particles/8500 cells) in subsequent experiments.

In response to both extracellular (extrinsic) and intracellular (intrinsic) death signals, caspase 3 is activated by upstream caspases through proteolytic cleavage and, in turn, cleaves several substrates, executing apoptosis. The ratio between cleaved and total caspase 3 was reduced after 24-hour incubation of MCF7 cells with EVs-cases, as compared with vehicle and EVs-controls (*Figure 2A*). After treating MCF7 cells with Dox, the trend became statistically significant (*Figure 2B*).

**Figure 2:**
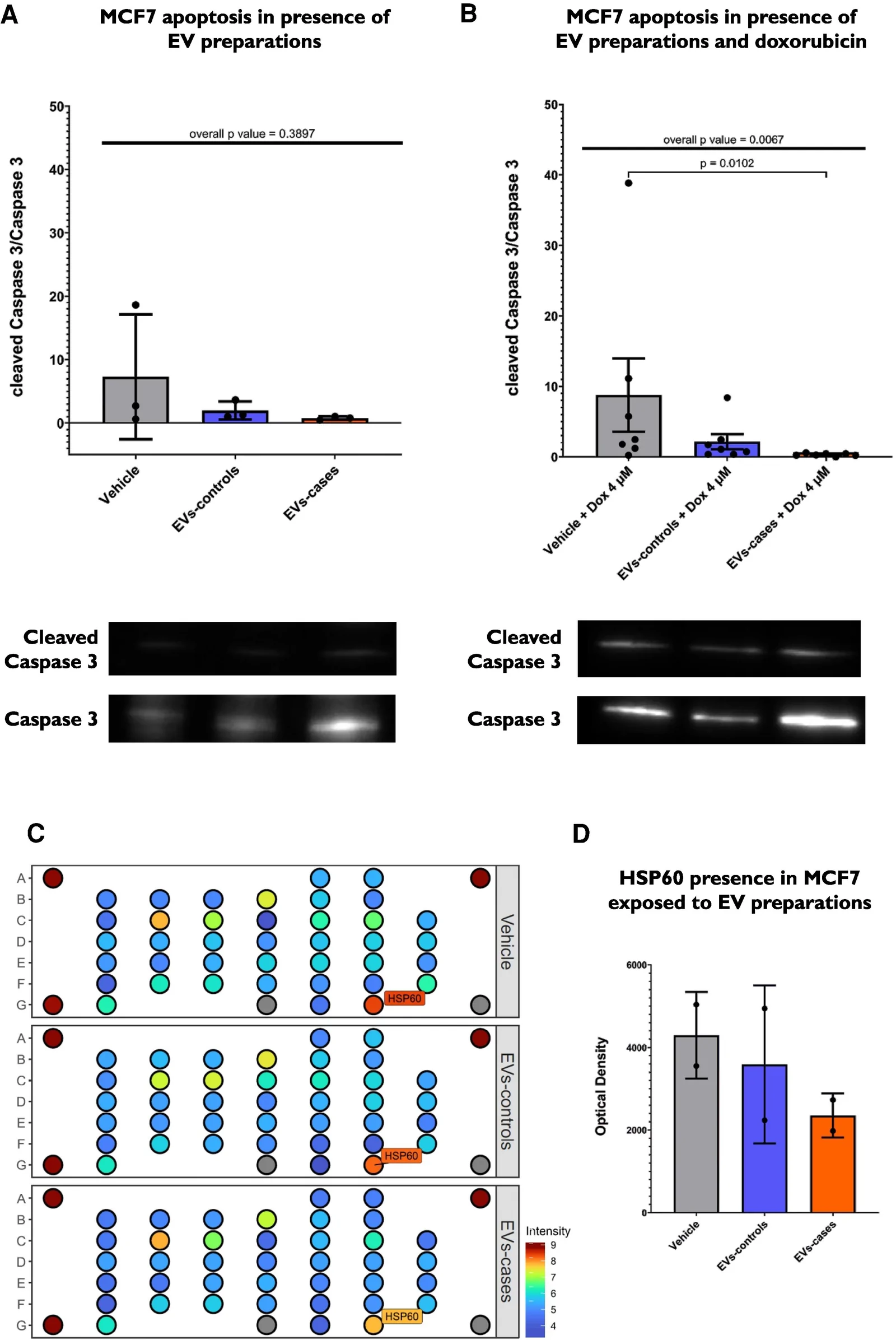
Apoptosis and phosphokinase expression in MCF7 cells treated with EVs-controls or EVs-cases. (*A*) Caspase 3 activation, as assessed by the ratio between cleaved and total caspase 3, in MCF7 cells incubated for 24 h with vehicle (grey), EVs-controls (blue), or EVs-cases (orange). EV preparations were used at a final concentration of 1 × 10^8^/8500 cells. Data are presented as mean and SD and were compared by one-way ANOVA with Tuckey’s *post hoc* test. *n* = 3. (*B*) Caspase 3 activation, as assessed by the ratio between cleaved and total caspase 3, in MCF7 cells incubated with Dox 4 µM for 24 h after 24 h preincubation with vehicle (grey), EVs-controls (blue) or EVs-cases (orange). EV preparations were used at a final concentration of 1 × 10^8^/8500 cells. Data are presented as mean and SD and were compared by Kruskal–Wallis test with Dunn’s *post hoc* test. *n* = 7. (*C*) Representation of the phosphokinase array after 24 h incubation of MCF7 cells with vehicle (top), EVs-controls (middle), or EV-cases (bottom). The expression levels of the target proteins are colour-coded according to the scale shown at bottom right, based on the mean intensity of 2 independent replicates. EV preparations were used at a final concentration of 1 × 10^8^/8500 cells. (*D*) Optical density of the HSP60 signal in the phosphokinase array (mean and SD, *n* = 2). EV, extracellular vesicle.

Insights into the pathways underlying the resistance to apoptosis elicited by EVs-cases were obtained from the screening of the phosphorylation status of a panel of intracellular kinases and related proteins. The cytosolic accumulation of HSP60 was previously reported to enhance breast cancer cell susceptibility to apoptosis.^20,21^ HSP60 was less abundant in whole MCF7 cell lysates after exposure to EVs-cases than EVs-controls (*Figure 2C and D*), while levels of mitochondrial HSP60 did not differ between the two groups (see Supplementary material online, *Figure S5*). Therefore, reduced levels of cytosolic HSP60 may have accounted for blunted apoptosis upon incubation with EVs-cases.

Transcription and spliceosome regulation were differentially represented cellular processes in the proteomes of EVs-cases vs. EVs-controls. Quantitative polymerase chain reaction (qPCR) screening of the expression of several genes encoding transcription and spliceosome regulators indicated that *AQR* and *NR3C1* were differentially expressed in MCF7 cells incubated with EVs-cases or EVs-controls, but this was not confirmed by targeted qPCR (see Supplementary material online, *Figure S6*).

As compared with vehicle, MCF7 cells treated with EVs-controls, but not EVs-cases, displayed a significant increase in ATP-linked respiration and proton leak rate (see Supplementary material online, *Figure S7B* and *C*). Other parameter of energy metabolism and ROS production were not significantly different between vehicle, EVs-cases and EVs-controls (see Supplementary material online, *Figure S7A* and *S7D*–*F*).

Proliferation of MCF7 cells in response to vehicle, EVs-cases, or EVs-controls was evaluated by quantifying EdU incorporation and anaphase/prophase distribution, as detected by aurora B kinase immunostaining. Both proliferation markers were variable across replicates and conditions, without any significant difference (see Supplementary material online, *Figure S8*).

#### Effects of HTC116 and A549 cells

Next, we sought to validate the observations in MCF7 cells with another 2 human cell lines from colon and lung cancer. It is noteworthy that these were the most represented malignancies among the cases we selected for this study. As shown in *Figure 3*, EVs-controls and EV-cases did not differently affect viability, apoptosis and expression of *AQR* in HCT116 and A549 cells. In contrast, levels of *NR3C1*, which encodes glucocorticoid receptor, in A549 cells were lower upon exposure to EVs-cases than to EVs-controls.

**Figure 3:**
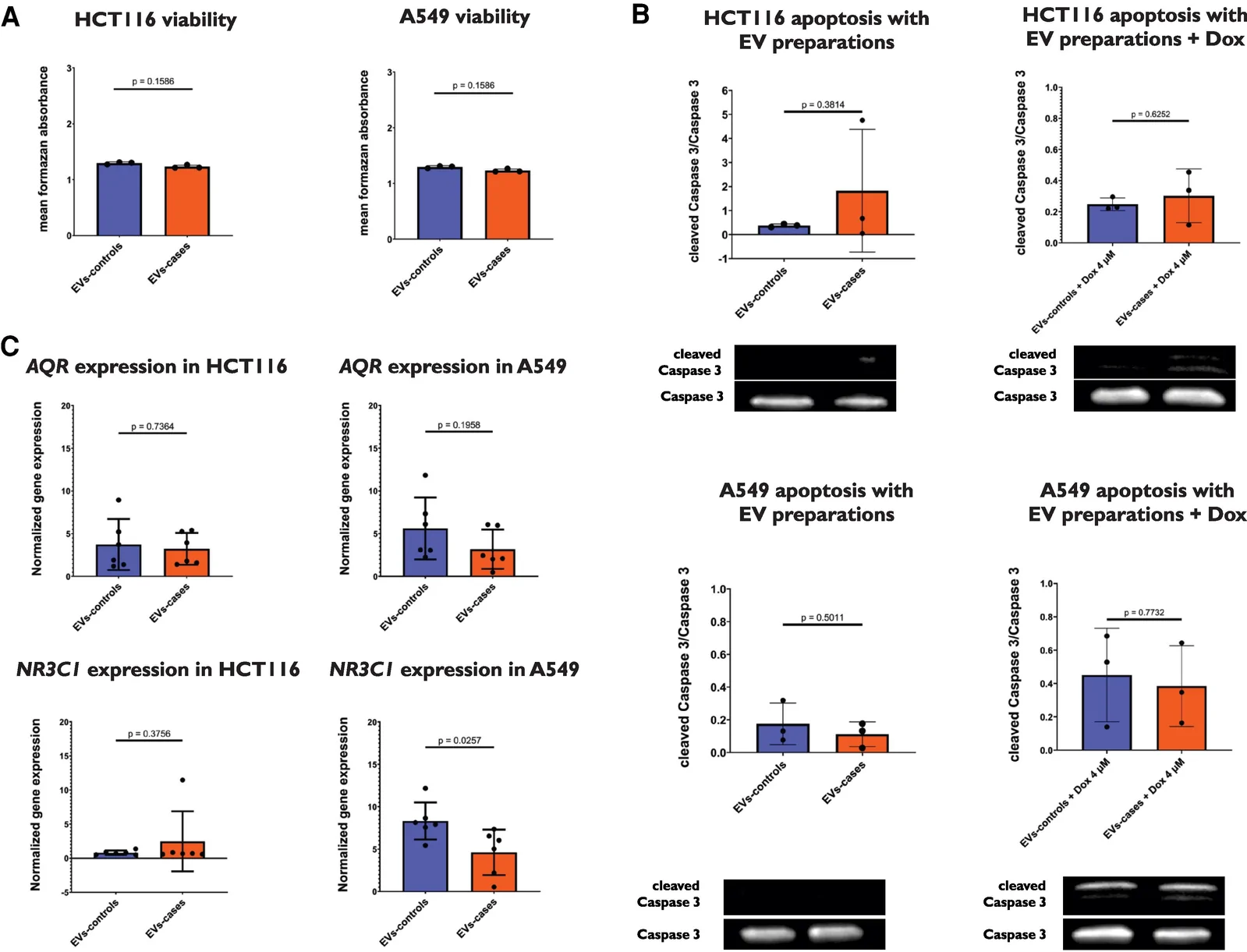
Viability, apoptosis, and *AQR* and *NR3C1* expression in HCT116 and A549 cells treated with EVs-controls or EVs-cases. HCT116 and A549 cells were treated for 24 h with EVs-controls (blue) or EVs-cases (orange) at a final concentration of 1 × 10^8^/8500 cells. (*A*) Viability of HCT116 (left panel) and A549 (right panel) cells after 24 h incubation with EVs-controls (blue) or EVs-cases (orange). Data are presented as mean ± SD and were compared by Student’s *t*-test. *n* = 3. (*B*) Caspase 3 activation, as assessed by the ratio between cleaved and total caspase 3, in HCT116 and A549 cells incubated with EV preparations for 24 h (left panels), or with Dox 4 µM for 24 h after 24 h preincubation with EVs-controls (blue) or EVs-cases (orange) (right panels). Data are presented as mean ± SD and were compared by Student’s *t*-test. *n* = 3. (*C*) Gene expression of *AQR* and *NR3C1* in HCT116 cells (left graphs) and A549 cells (right graphs) after 24 h incubation with EVs-controls (blue) or EVs-cases (orange). Data are expressed as mean normalized expression over reference genes (2^−ΔCq^) ± SD. *n* = 6.

## Discussion

This investigation shows that plasma EV-enriched fractions from patients with HFrEF and incident cancer have a distinctive protein signature, as compared to plasma EV-enriched fractions from patients who had HFrEF, but no cancer (*Graphical abstract*). Moreover, circulating EV fractions from patients with HFrEF and incident cancer might modulate some aspects of cancer cell biology. These results lend support to the hypothesis that EV-mediated molecular changes anticipate and prime cancer development in HFrEF. Nevertheless, the link between EVs and cancer in HFrEF in only preclinical and has to be confirmed.

HF and cancer, two of the leading causes of death worldwide, are interconnected within a complex network of common risk factors and pathophysiological pathways. A growing body of evidence, from epidemiological studies^22,23^ as well as preclinical models,^6,7^ indicates that HF promotes cancer growth.^24^ This may be due to several mechanisms. Germane to this investigation is the recent demonstration that, in a mouse model of HFrEF, cardiac cell-derived EVs induce proliferation of cancer cells and accumulation of cancer-tolerant macrophages at tumour sites.^6,10^

The importance of EVs in the pathogenesis of cardiovascular diseases is established.^25^ Isolation and analysis of EVs from peripheral blood can offer major insights in this regard.^26^ The comparison of the cargos of circulating EVs from HF patients and healthy controls has revealed DEPs related to proliferation, inflammation, and metabolism.^27^ On the other hand, EVs may also mediate inter-organ communications leading to extra-cardiovascular complications in patients with HF, and cancer-specific circulating EV profiles have been described. Proteomic analysis of plasma EVs highlighted significant differences between patients with breast cancer, patients with benign breast lesions, and healthy women.^28^

By taking advantage of the characterization of cancer in GISSI-HF, we were able to explore the protein content of HFrEF circulating EV-enriched fraction with respect to cancer incidence. To our knowledge, this has never been done so far.

Two features make GISSI-HF uniquely suitable for the present study. First, the enrolled HF patients underwent venous blood collection at baseline. Second—unlike in most other HF trials—new-onset tumours were captured in case report forms and the investigators were asked to enter information about the type of tumour.^12^ Hence, we could select a subgroup of GISSI-HF participants with incident malignant tumours, *i.e.* cancer, and match them with other patients who did not present any tumour during follow-up. Since experimental data on the connection between HF and cancer have mainly been obtained with HFrEF mouse models,^5^ we focused on this HF phenotype. Matching minimized the possible confounding effect of sex, age and HF severity, as determined by NYHA class. Moreover, the risk of surveillance bias was controlled by further matching by duration of follow-up.

Against this background, we identified a protein profile of circulating EV preparations that tracks with incident cancer in HFrEF. The DEPs between EVs-cases and EVs-controls were related to a range of biological processes implicated in tumour progression.

To verify that plasma EV preparations from patients with HFrEF and incident cancer do have activities on cancer cells, we performed additional proof-of-principle experiments *in vitro*.

Assessment of caspase 3 activation in MCF7 cells indicated that EVs-cases may induce resistance to apoptosis, possibly via regulation of non-nuclear HSP60.^29^ However, EV-cases did not modulate apoptosis differently from EV-controls in HCT116 and A549 cells. On the other side, we did not observe any effect of EV-cases on the levels of *AQR* and *NR3C1* in MCF7 and HCT116 cells, but *NR3C1* expression was significantly decreased in A549 cells after incubation with EV-cases. Intriguingly, it was previously reported that activation of glucocorticoid receptor by glucocorticoids halts the proliferation of A549 cells.^30,31^ Downregulation of *NR3C1* might prevent this cancer-limiting effect.

These data hint at some activities of the HFrEF plasma EV-enriched fraction on cancer cells, which however would be variable and cancer type-dependent. Further research is needed to understand whether and which tumour-promoting signals are carried by circulating EVs in HFrEF. In particular, we found that EVs-cases were enriched for inflammatory/immune mediators (IL6ST, CHIT1, and CPN1), which could therefore be the focus of future investigations.

We acknowledge that our work has limitations. The choice of the cases was partly dictated by the availability of plasma and this might have introduced a selection bias. Furthermore, this led to a small sample size undermining the proteomic analysis. The plasma aliquots we used had been stored at −80°C for years, although the quality of the GISSI-HF plasma samples was good and no challenges were encountered during EV fraction isolation and subsequent protein extraction. We cannot exclude the possible effect of rosuvastatin and n-3 polyunsaturated fatty acids (interventions in GISSI-HF), as well as of HF therapies, on cancer growth. The polymer-based precipitation method we employed co-isolated non-vesicular extracellular particles and soluble plasma proteins and lipoproteins, which could influence cancer cell biology in parallel with EVs.

It is also true that collection, processing, and storage were rigorous in the well-organized context of a clinical trial, preventing flaws in the pre-analytical phase that commonly jeopardize omics studies with patient-derived material. Although impure, our plasma preparations mainly contained EVs. Furthermore, the absolute numbers of DEPs between cases and controls were very similar to those obtained by other authors by proteomic analysis of EVs in fresh blood samples from mice with or without HFrEF.^6,10^

The experiments *in vitro* give cues that circulating EV-enriched fractions from patients with HFrEF and incident cancer are biologically active on cancer, but should be considered hypothesis-generating. They were carried out with cell lines, corresponding only to some of the tumour types that had occurred in cases. Moreover, the main results were not reproducible across these lines. Additional, dedicated investigations are needed to dissect whether circulating EVs from patients with HFrEF have different activities on normal vs. cancer cells, as well as to understand to which extent and how they affect cancer biology. The source of cancer-facilitating EVs and the interplay with sex hormones also remain to be determined.

In conclusion, we provide novel evidence that a proteomic signature of the circulating EV-enriched fraction is associated with incident cancer in human HFrEF, and we set a line of research into the pathways underlying this association.

## Supplementary Material

oeag180_Supplementary_Data

## Data Availability

Mass spectrometry data were deposited to ProteomeXchange via PRIDE14 and are publicly available under the accession number MSV000095415. The other data underlying this article will be shared on reasonable request to the corresponding author.
