# Supplementary material for: Circulating extracellular vesicle-enriched fraction in heart failure with reduced ejection fraction and incident cancer
Source: Eur Heart J Open. 2026 Oct 26;6(6):oeag180. doi: 10.1093/ehjopen/oeag180 (PMC13739045; doi:10.1093/ehjopen/oeag180)
Supplement: Supplementary file 1 [file oeag180_supplementary_data.zip › EVs in HFrEF with cancer_supplemental_R1_clean.docx]

**Circulating extracellular vesicle-enriched fraction in heart failure with reduced ejection fraction and incident cancer**

Bianconi E, De Palma A, Perico D, Bruno S, Fumagalli F, Nicolis E, Rulli E, Ceresa D, Costa A, Vitale C, Latini R, Mauri PL, Ameri P

**Supplemental material**

Full methods: pages 2-10

Table S1: page 11

Table S2: page 12

Table S3 legend: page 13

Table S4 legend: page 13

Figure S1: page 14

Figure S2: page 15

Figure S3: page 16

Figure S4: page 17

Figure S5: page 18

Figure S6: page 19

Figure S7: pages 20-21

Figure S8: pages 22-23

Bibliography: pages 24-25

**Full methods**

**Selection of plasma samples from patients**

GISSI-HF trial was a randomised, double-blind, placebo-controlled trial testing the efficacy of n-3 polyunsaturated fatty acids in improving morbidity and mortality in patients with HF ^1^. Peripheral venous blood was collected from the study participants at baseline, aliquoted, and stored for future research. Moreover, information on cancer status was accurately recorded ^2^.

We identified the participants in the trial without cancer at the time of enrolment and with available plasma samples, and we divided them in two groups: those who were diagnosed with cancer or died from cancer at follow-up (cases), and those who remained cancer-free (controls). We excluded patients with LVEF ≥40% and/or haematological malignancies, since preclinical investigations have linked HFrEF with solid cancer. Unsurprisingly, the number of cases was much lower than the number of controls. Thus, we matched 1:1 cases to controls, based on age, sex, New York Heart Association (NYHA) class, and follow-up duration.

**Extracellular vesicle isolation and characterization**

EVs from cases and controls were extracted from 200µL of plasma for each subject by using ExoQuick® ULTRA kit (System Biosciences, Palo Alto, CA, USA), according to the manufacture’s protocol. This polymer based precipitation method includes a post-precipitation clean-up step, where, after incubation with ExoQuick reagent for 30 min at 4 °C and then 10-min centrifugation at 3000 × g, the pellets are resuspended in 500µL of ExoQuick Ultra buffer and loaded to a clean-up resin column to lower plasma protein contamination. Only part of the plasma isolated EVs was processed for the proteomic analysis (see below). The leftovers of each single sample were pooled together to form a batch of EVs from cases and batch of EVs from controls. The concentration of both pools was measured as particles/mL by performing nanoparticles tracking analysis (NTA) with Nanosight NS300 (Malvern Panalytical, Malvern, United Kingdom). Samples were diluted 1:1000 with Phosphate Buffer Solution (PBS). The screen gain was set as 1,0 in capture and 10 in tracking and 5 captures of 60 seconds each. Videos were then processed to generate size distribution (hydrodynamic diameter) and concentration (particles/mL) of both preparations.

Then, the evaluation of EV markers ^3^ was performed by western blot analysis. Samples were lysed in RIPA Buffer (Thermo Fisher Scientific, San Josè, CA, USA) supplemented with protease and phosphatase inhibitor cocktail (Cell Signaling Technologies - CST, Danvers, Massachusetts, USA) and the protein contents were measured with Pierce BCA Protein Assay Kits (Thermo Fisher Scientific) following the manufacturer’s instruction. Samples were separated through a tris-glycine gel (8-16%; Bio-Rad, Hercules, California, USA), transferred onto a methanol activated PVDF membrane (Immobilon PVDF Membrane, Merck, New York, USA) and incubated with anti-CD9 (ab236630, diluted 1:2000; Abcam, Cambridge, UK), anti-ALIX (ab186429, diluted 1:1000; Abcam), anti-FLOTILLIN-1 (ab133497, diluted 1:10,000; Abcam) and anti-TSG101 (T5701, diluted 1:1000; Sigma Aldrich); primary antibodies were detected by incubation of the membranes with the anti-rabbit HRP-conjugated secondary antibodies (CST). Western Blot bands were visualized with Clarity Western ECL Substrate (Bio-Rad).

**Extracellular vesicle protein extraction, and enzymatic digestion**

After isolation with Exoquick ULTRA kit, EVs were digested to perform the subsequent Liquid Chromatography-Tandem Mass Spectrometry (LC-MS/MS) analysis. Protein concentration in EVs was determined with a SPN^TM^-Protein assay kit (G-Biosciences, St. Louis, MO, USA). Each protein mixture (50 ug) was suspended in 0.1M NH_4_HCO_3_ pH 7.9, treated with RapiGest^TM^ SF reagent (Waters Co, Milford, MA, USA) at the final concentration of 0.25% (w/v), and incubated while stirring at 95°C for 15 min. The digestion was carried out on each sample by adding Trypsin/Lys C Mix, Mass Spec Grade (Promega Inc., Madison, WI, USA), at an enzyme/substrate ratio of 1:25 (w/v) overnight at 37°C in 0.1M NH_4_HCO_3_ pH 7.9 buffer with 10% CH_3_CN. The enzymatic reaction was stopped by addition of 0.5% Trifluoroacetic acid (TFA) (Sigma-Aldrich Inc., St. Louis, MO, USA) and incubated at 37°C for 45 min to complete the RapiGest acid hydrolysis. The water-immiscible degradation products were removed by centrifugation at 13,000 rpm for 10 min. The tryptic digest mixtures were desalted using Pierce^TM^ C-18 spin columns (Life Technologies, Segrate, Italy), according to the manufacturer protocol and were resuspended in 0.1% formic acid (Sigma-Aldrich Inc.) in water (LC-MS Ultra CHROMASOLV^TM^, Honeywell Riedel-de HaenTM, Muskegon, MO, USA) at a concentration of 0.1 µg/µL.

**LC-MS/MS Analysis**

Peptide mixtures were analyzed using Eksigent nanoLC-Ultra® 2D System (Eksigent, part of AB SCIEX Dublin, CA, USA) combined with cHiPLC-nanoflex system (Eksigent) in trap-elute mode. Briefly, 0.8 µg of proteins of each condition in technical duplicates were injected on the cHiPLC trap (200 µm x 500 µm ChromXP C18-CL, 3 µm, 120 Å, Eksigent, part of AB SCIEX Dublin, CA, USA) and run the loading pump in isocratic mode with 0.1% formic acid in water for 10 minutes at a flow of 3 µL/min. The automatic switching of cHiPLC ten-port valve then eluted the trapped mixture on a nano cHiPLC column (75 µm x 15 cm ChromXP C18-CL, 3 µm, 120 Å (Eksigent) through a 95 minutes gradient of eluent B (eluent A, 0.1% formic acid in water; eluent B, 0.1% formic acid in acetonitrile) at a flow rate of 300 nL/min. The eluted peptides were directly analyzed on an Orbitrap Exploris 120 mass spectrometer (Thermo Fisher Scientific) equipped with EASY-Spray ion source (Thermo Fisher Scientific). Easy spray was achieved using an EASY-Spray Emitter (Thermo Fisher Scientific) (nanoflow 7 µm ID Transfer Line 20 µm x 50 cm) held to 1.6 kV, while the ion transfer capillary was held at 220°C. Data-dependent acquisition (DDA) was performed, acquiring precursor ions in m/z range of 375–1,250 with a resolution (at m/z 200) of 60,000 FWHM (full width at half maximum). Precursor fragmentation was carried out at a resolution (at *m/z* 200) of 15,000 FWHM, using higher-energy collisional dissociation (HCD) method with normalized collision energy (NCE) of 30 eV and a dynamic exclusion of 20 sec. MS and MS/MS data were acquired in profile and centroid mode, respectively, using positive polarity and isotope exclusion was on. Isolation width was set at 2 m/z and first mass was set to 120 *m*/*z*. Mass spectrometer scan functions and high-performance liquid chromatography solvent gradients were controlled by the Xcalibur data system version 4.4 (Thermo Fisher Scientific) and Eksigent Control Software version 4.3 (Eksigent), respectively.

**Proteomic data analysis**

All raw data generated were searched in *Homo* *sapiens* proteome database downloaded on March 2021 from Uniprot ([www.uniprot.org](http://www.uniprot.org)) using the Sequest HT search engine contained in Proteome Discoverer software, version 2.5 (Thermo Fisher Scientific). MSPepSearch node has been inserted in the processing workflow before Sequest HT to perform a first spectral library search (NIST Human Orbitrap HCD library, 1127970 spectra, September 2016). The following criteria were used for the identification of peptide sequences and related proteins: mass tolerances of ± 10 ppm for precursor ions and ± 0.02 Da for fragment ions; trypsin as enzyme with maximum of two missed cleavage tolerance; methionine oxidation as variable modification. Peptide-spectrum matches were validated in Proteome Discoverer v2.5 using Percolator with a target–decoy strategy, applying a false discovery rate (FDR) threshold of 1% (q ≤0.01). Protein inference and validation were performed using the Protein FDR Validator with 1% FDR and minimum two high-confidence peptides per protein ^4^. Label free quantitation (LFQ) of proteins was conducted by setting a non-nested study with biological and technical replicates, using the spectrum file recalibration and Minora algorithm with precursor ion peak intensity of unique peptides for abundance calculation when 60% of replicate features was reached. A pairwise ratio-based approach was set for the comparison of normalized protein abundances among the examined sample groups. Protein grouping and strict parsimony principle were also applied.

Differential expressed proteins (DEPs) were identified if the log2 fold change (FC) was ≥ │1.5│and the p-value <0.001 (Benjamini- Hochberg correction). Heatmap and Principal Component Analysis (PCA) were obtained using JMP v15 (SAS Institute Inc.).

The clustering analysis was performed using the Euclidean Ward method for distance and aggregation of all proteins. The MS data have been deposited to the ProteomeXchange Consortium via the PRIDE ^5^ partner repository with the dataset identifier <ftp://MSV000095415@massive.ucsd.edu>.

The cellular component enrichment of proteins in the examined conditions and the comparison versus the Vesiclepedia database were achieved using FunRich version 3.1.3, open access standalone software download from http://www.funrich.org/ (accessed on 10 June 2025) ^6^.

Utilizing the identified DEPs, we constructed a protein–protein interaction (PPI) network comprising 134 nodes and 170 edges. This network was built using the STRING database ^7^, which considers only experimentally and database-defined PPIs with a score >0.4. The resulting sub-network was visualized and analysed through Cytoscape v. 3.10.1 and its plugins ^8^.

To enhance interpretation, proteins were organized into functional modules with the support of STRING enrichment, applying default settings. Node colours represent the condition under examination in the pairwise comparison.

**Cellular experiments**

EVs isolated from patients with or without incident cancer and diluted in ExoQuick ULTRA buffer (hereafter, vehicle) were pooled together to form two batches (*i.e.* incident cancer and no cancer).

The effects of EVs from controls and cases were tested on the human breast adenocarcinoma cell line MCF7, provided by the Interlab Cell Line Collection of IRCCS Ospedale Policlinico San Martino (Genova, Italy). The human breast adenocarcinoma cell line MCF7 was provided by the Interlab Cell Line Collection (ICLC) of IRCCS Ospedale Policlinico San Martino (Genova, Italy). Cells were maintained in RPMI 1640 medium, supplemented with 10% of Fetal Bovine Serum (FBS), 2% L-Glutamine, and 1% penicillin-streptomycin (all reagent were provided by Euroclone, Milano, Italy), at 37°C with 95% of humidity and 5% of CO_2_. Cells were splitted 1:3 to 1:5 when confluence reached 70-80%.

The effects of EVs from cases and controls were assessed by incubating MCF7 cells for 24h. For these experiments, cells were seeded at 25,000 cells/cm^2^ and kept in RPMI +1% FBS.

Since the EVs were diluted in the ExoQuick ULTRA buffer (hereafter referred to as vehicle), the latter was included as control condition in all experiments, at the same volume as the one for EVs.

**Assessment of cellular viability**

Twenty-four hours after seeding, MCF7 cells were incubated with buffer or with EVs, at concentrations ranging from 1 × 10^4^ to 1 × 10^9^ particles/8,500 cells, for 24h and viability was determined with the MTS- based assay “Cell Titer 96 AQueous One Solution Cell Proliferation Assay” (Promega Inc.), following the manufacturer’s recommendations.

Since no effect of EVs was observed on cell proliferation at the evaluated concentrations, the one of 1 × 10^8^ particles/8,500 cells was used in the subsequent experiments.

**Assessment of cellular proliferation**

MCF7 were seeded in 8-well chamber slides (Lab-Tek II Chamber slide w/cover; Thermo Fisher Scientific) in standard conditions for 24h; then, cells were incubated for 24h with vehicle or EVs. To assess DNA duplication, treated cells were incubated for 3h with EdU at the final concentration of 10 µM and then fixed with formalin 10% in PBS for 15 min at room temperature (RT). EdU incorporation was revealed with Click-iT EdU Alexa 555 HCS (Thermo Fisher Scientific) following the manufacturer’s instructions. In addition, the quantification of MCF7 in anaphase and prophase was performed by Aurora B kinase immunostaining (ab2254, diluted 1:50; Abcam, Cambridge, UK), counterstained for cytoskeletal actin with Phalloidin AlexaFluor 555 conjugated (Thermo Fisher Scientific) and 4',6-diamidino-2-phenylindole (DAPI, Thermo Fisher Scientific) for nuclei visualization. Images of both EdU and Aurora B kinase staining were acquired with Axio Imager M2 (Zeiss, Oberkochen, Germany). EdU positive cells were counted with ImageJ and expressed as the mean percentage of positive cells (n=3 ± DS) while Aurora B kinase immunostained cells were counted manually in order to identify anaphases and prophases and expressed as the ratio anaphase or prophase cells/total nuclei (n=3; mean ± SD).

**Assessment of apoptosis**

MCF7 were seeded in standard conditions in 12 well plates (Euroclone) for 24h then incubated for additional 24h with EVs or vehicle. In addition to the basal experiment (*i.e.* cells incubated with EVs from controls and cases or vehicle), EVs or vehicle pre-incubated MCF7 were induced to undergo apoptosis with an 24h-long incubation with doxorubicin (Dox) 4 µM ^9^. At the end of the stimulation, cells were lysed with EuroGOLD TRiFast (Euroclone) and lysates were processed following the manufacturer’s recommendations in order to extract total proteins.

Samples were separated through a tris-glycine gel (8-16%, Thermo Fisher Scientific), transferred onto a nitrocellulose membrane (Thermo Fisher Scientific), and incubated with anti–caspase3, anti–cleaved caspase3 diluted 1:1000 (#9664 and #14220, CST) and horseradish peroxidase (HRP)-conjugated anti-GAPDH (#8884; CST). Apart from the latter, primary antibodies were detected by incubation of the membranes with the proper HRP-conjugated secondary antibodies (CST). Western blot bands were visualized with Clarity Western ECL Substrate (Bio-Rad) and quantified by densitometry with ImageJ-Fiji analysis tool. Experiments with EVs or vehicle was repeated 3 times while Dox-induction ones 7 times. Data were expressed as ratio Cleaved-to-total Caspase3.

**Mitochondrial metabolic analysis**

Mitochondrial bioenergetic characteristics of MCF7 treated with vehicle, EVs from controls or cases were determined in an XF24 extracellular flux analyzer (Seahorse Biosciences, Agilent Technologies, Santa Clara, CA) in 16-well SeaHorse XFp Cell miniplate (Seahorse Bioscience). At the end of the treatment, the cell medium was replaced by XF Base Medium supplemented with 10 mM glucose, 10 mM pyruvic acid and 1 mM L-glutamine and incubated in absence of CO_2_ for 1 h before the experiment. Then, the Oxygen Consumption Rate (OCR) analyses were performed in a Seahorse Bioscience XF flux analyzer according to the manufacturer protocol. Extracellular fluxes were recorded after serial injection of Oligomycin, FCCP (carbonyl cyanide-p trifluoromethoxy phenylhydrazone), and antimycin A/rotenone diluted in XF assay medium at final concentrations of 1.5 μM, 1.0 μM, and 0.5 μM, respectively. The whole experiment was repeated 4 times.

**Reactive oxygen species production**

The amount of reactive oxygen species (ROS) in MCF7 after treatments with vehicle, EVs from cases or controls were measured by adding the CellROX® Deep Red Reagent (Thermo Fisher Scientific) at a concentration of 5 µM directly to the culture medium. After a 30 minutes long incubation at 37°C, cells were collected, resuspended in PBS and analyzed with CytoFLEX (Beckman-Coulter, Pasadena, California, U.S.A.). Data from 5 experiments were analyzed by FlowJo 10.10 software.

**Phospho-kinase quantification**

MCF7 cells were seeded in 12-well plates (Euroclone) and, after 24h in standard conditions, exposed for 24 hours EVs from cases, controls or vehicle in order to assess phospho-kinase expression with Proteome Profiler Human Phospho-Kinase Array Kit (R&D systems, Minneapolis, MN, USA), in accordance with manufacturer’s protocol. The whole experiment was repeated twice.

**HSP60 localization with confocal microscopy**

MCF7 were seeded in 8-well chamber slides (Lab-Tek II Chamber slide w/cover, Thermo Fisher Scientific) in standard conditions for 24h and then incubated for 24h with vehicle or EVs. Cells were fixed in 10% formalin in PBS and immunostained with anti-HSP60 monoclonal antibody (Thermo Fisher Scientific) and with rabbit polyclonal antibody against TOM20 (CST). Mitochondrial reticulum and HSP60 fluorescence were detected on the laser scanning spectral confocal microscope TCS SP2 AOBS (Leica, Heidelberg, Germany), using appropriate lasers. Images were acquired through a HCX PL APO CS 40 ×/1.25 oil UV objective, the pinhole was set to 1 Airy unit and the imaging was performed at 1024 × 1024 pixels per image. Co-localization was expressed as percent of above-background HSP60-containing pixels that overlapped above-background TOM20-positive ones, and TOM20-containing pixels that overlapped HSP60-positive ones, with background threshold set by the Costes’ method ^10^.

**Gene expression analysis**

MCF7 cells were seeded in 12-well plates (Euroclone) and exposed for 24 hours EVs from cases, controls or vehicle. Total RNA was collected with EuroGOLD TriFast™ reagent (Euroclone) and purified with Quick-RNA Minipreps (Zymo Research, Irvine, CA, USA) following the manufacturer’s recommendations. RNA quantity and quality assessment were assessed with NanoDrop2000 (Thermo Fisher Scientific). Total RNA was reverse transcribed with iScript Advanced cDNA Synthesis kit for RT-qPCR (Bio-Rad).

At first, the expression of a set of markers for “regulation of gene expression” and “spliceosome regulation” was screened with company-designed primers and SYBR-dye based chemistry assays (n=3; Bio-Rad). Then, *AQR (n=7)*, *NR3C1 (n=5)* and *VDR (n=2)* normalized relative expressions were assessed with company-designed probes (Integrated DNA Technologies IDT, Coralville, Iowa, U.S.A.) and Luna Universal Probe qPCR Master Mix 2X (New England Biolabs, Ipswich, Massachusetts, U.S.A.). Gene markers were listed in **Table S1**. Amplifications were performed with CFX Touch (Bio-Rad) and relative gene expression was calculated and analyzed with CFX Manager software (Bio-Rad).

**Statistical analysis**

Data from the cell experiments were analysed by one-way ANOVA with Tuckey’s *post hoc* test or Kruskal-Wallis test with Dunn’s *post hoc* test, depending on the distribution, by using Graph Pad PRISM 10. Statistical significance was set at p <0.05.

Gene expression data were handled and analysed with CFX Manager software (Bio-Rad).

**Supplemental Table S1. Assays used for qPCR**

| **Gene name** | **Assay ID** | **Fluorophore** | **Manufacturer** |
| --- | --- | --- | --- |
| *ACTB** | qHsaCED0036269 | SYBR | Bio-Rad |
|  | Hs.PT.39a.22214847 | FAM | IDT |
| *AKR1B1* | qHsaCID0023127 | SYBR | Bio-Rad |
| *AQR* | qHsaCID0016538 | SYBR | Bio-Rad |
|  | Hs.PT.58.27823847 | FAM | IDT |
| *AR* | qHsaCID0007155 | SYBR | Bio-Rad |
| *AREG* | qHsaCID0037067 | SYBR | Bio-Rad |
| *CTNNB1* | qHsaCED0046518 | SYBR | Bio-Rad |
| *ESR1* | qHsaCED0042641 | SYBR | Bio-Rad |
| *ESR2* | qHsaCED0044944 | SYBR | Bio-Rad |
| *HDAC1* | qHsaCID0021964 | SYBR | Bio-Rad |
| *IPO8** | qHsaCED0056515 | SYBR | Bio-Rad |
|  | Hs.PT.58.40532361 | FAM | IDT |
| *NOTCH1* | qHsaCID0011825 | SYBR | Bio-Rad |
| *NR3C1* | qHsaCED0043506 | SYBR | Bio-Rad |
|  | Hs.PT.58.27480377 | FAM | IDT |
| *RARB* | qHsaCID0009168 | SYBR | Bio-Rad |
| *RBM22* | qHsaCED0042547 | SYBR | Bio-Rad |
| *RELA* | qHsaCED0046951 | SYBR | Bio-Rad |
| *THRB* | qHsaCID0012393 | SYBR | Bio-Rad |
| *TP53* | qHsaCID0013658 | SYBR | Bio-Rad |
| *VDR* | qHsaCID0023190 | SYBR | Bio-Rad |
|  | Hs.PT.58.20635582 | FAM | IDT |

* Reference gene

**Supplemental Table S2. Characteristics of controls and cases after matching for age, sex, NYHA class, and follow-up duration.**

|  | **Controls N=30** | **Cases N=30** | **Overall N=60** |
| --- | --- | --- | --- |
| **Died from cancer – n (%)** | - | 16 (53.3) | 16 (53.3) |
| **Hospitalized from cancer – n (%)** | - | 28 (93.3) | 28 (93.3) |
| **Type of cancer** |  |  |  |
| Gastric | - | 11 (36.7) | 11 (36.7) |
| Lung | - | 6 (20.0) | 6 (20.0) |
| Breast | - | 1 (3.3) | 1 (3.3) |
| Prostate | - | 3 (10.0) | 3 (10.0) |
| Brain | - | 1 (3.3) | 1 (3.3) |
| Skin | - | 1 (3.3) | 1 (3.3) |
| Uro-genital | - | 4 (13.3) | 4 (13.3) |
| Others | - | 3 (10.0) | 3 (10.0) |
| **Age** |  |  |  |
| Mean (SD) | 65.6 (7.6) | 66.1 (7.7) | 65.9 (7.6) |
| **Sex** |  |  |  |
| Male | 26 (86.7) | 26 (86.7) | 52 (86.7) |
| Female | 4 (13.3) | 4 (13.3) | 8 (13.3) |
| **NYHA class** |  |  |  |
| II | 21 (70.0) | 21 (70.0) | 42 (70.0) |
| III+IV | 9 (30.0) | 9 (30.0) | 18 (30.0) |
| **LVEF** |  |  |  |
| Mean (SD) | 31.7 (0.6) | 31.1 (6.0) | 31.4 (5.9) |
| **Time in study (months)** |  |  |  |
| Mean (SD) | 44.5 (11.3) | 43.4 (12.5) | 44.0 (11.8) |

LVEF: left ventricular ejection fraction; NYHA: New York Heart Association.

**Supplemental Table S3. List of the 940 master proteins identified by label-free quantification.**

Controls are color-coded in blue and cases in orange. For each protein the following information is provided: Uniprot accession, description, gene name, peptides, peptide spectrum matches (PSMs), unique peptides, molecular weight (MW), calculated isoelectric point (pI), presence only in one group, and average abundances by biological replicates and each examined condition.

**Supplemental Table S4. List of DEPs between cases and controls.**

For each protein, the following information is provided: Uniprot accession, gene symbol, protein description, peptide spectrum matches (PSMs), unique peptides, molecular weight (MW), calculated isoelectric point (pI), log2 abundance ratio, average abundances by condition, and direction of change (upregulated in cases or controls).

**Supplemental Figure S1. Flowchart of case and control selection.**

LVEF: left ventricular ejection fraction; NYHA: New York Heart Association.

The figure was created with the free version of BioRender (www.biorender.org).

**Supplemental Figure S2.** **Characterization of the identified proteins as associated with EVs.**

A. Venn diagrams illustrating the overlap between the proteins detected in plasma of controls (blue) and cases (orange) and those recorded in the Vesiclepedia database. All gene entries from Vesiclepedia were downloaded and imported in Funrich (13,550 entries from 813 experiments).

B. Enrichment analysis of cellular components in controls (blue) and cases (orange). The left y-axis indicates the percentage of enriched genes for each cellular component and the right y-axis indicates the corresponding–log10 Benjamini-Hochberg corrected p values. Only gene ontology terms with a fold change >2 and a p value <1E-6 were considered.

**Supplemental Figure S3.** **Protein distribution and abundances in controls and cases.**

A. Venn Diagram showing the distribution of all quantified proteins in controls (blue) and cases (orange).

B. Box plot showing the distribution of average protein abundances by biological replicates in controls (blue) and cases (orange).

S**upplemental Figure S4. MCF7 cell viability upon exposure to EVs from controls or cases.**

Cell viability was assessed after incubating MCF7 cells for 24h with vehicle (grey), EVs from controls (blue), or EVs from cases (orange) at concentrations ranging from 1 x 10^9^ and 1 x 10^4^ per 8,500 cells. Data are presented as normalized viability over vehicle with violin plots, n=4.

**Supplemental Figure S5. Mitochondrial HSP60 localization in MCF7 cells upon exposure to EVs from controls or cases.**

Representative images of the co-localization of HSP60 with the mitochondrial protein TOM20 (A) and quantification of HSP60 in mitochondria (B) after incubating MCF7 cells for 24h with vehicle (grey), EVs from controls (blue), or EVs from cases (orange). EVs were used at a final concentration of 1 × 10^8^/8,500 cells. The fluorescence of mitochondrial HSP60 was measured in 1,100 cells per experimental condition; in the violin plots in (B), every dot represents the mean fluorescence of 10 cells.

**Supplemental Figure S6. Expression of regulators of transcription and spliceosome in MCF7 cells upon exposure to EVs from cases or controls.**

Gene expression of select genes involved in the regulation of transcription and spliceosome after incubating MCF7 cells for 24h with vehicle (grey), EVs from controls (blue), or EVs from cases (orange). EVs were used at a final concentration of 1 × 10^8^/8,500 cells.

A. Array, n=4

B-E. Targeted qPCR for *AQR* (B, n=7), *NR3C1* (C, n=5) and *VDR* (D, n=2).

Data are presented as mean ± SD.

**Supplemental Figure S7. Mitochondrial metabolism in MCF7 cells treated with plasma EVs from cases or controls.**

A-E. Extracellular flux analysis of energy metabolism in MCF7 cells treated for 24h with vehicle (grey), EVs-controls (blue), or EVs-cases (orange). EVs were used at a final concentration of 1 × 10^8^/8,500 cells. Data are presented as violin plots and were compared by one-way ANOVA with Tuckey’s *post hoc* test. n=4.

A. Basal respiration, expressed as oxygen consumption rate (OCR).

B. ATP-linked respiration, calculated as the difference between basal respiration and OCR recorded after adding ATP synthase inhibitor oligomycin.

C. Proton leak, calculated as the difference between OCR recorded after adding oligomycin and OCR recorded after adding the inhibitors of mitochondrial complexes I and III rotenone/antimycin A.

D. Maximal respiration, expressed as OCR after adding the uncoupling agent of oxidative phosphorylation FCCP

E. Spare capacity, calculated as the difference between maximal and basal.

F. ROS production in MCF7 cells treated for 24h with vehicle (grey), EVs from controls (blue), or EVs from cases (orange). EVs were used at a final concentration of 1 × 10^8^/8500 cells. Data are presented as violin plots and were compared by one-way ANOVA with Tuckey’s *post hoc* test. n=5.

**Supplemental Figure S8. MCF7 cell proliferation upon exposure to EVs from controls or cases.**

A and B. Percentage of EdU-positive cells (A) and representative images of EdU staining (B) after incubating MCF7 cells for 24h with vehicle (grey), EVs from controls (blue), or EVs from cases (orange). EVs were used at a final concentration of 1 × 10^8^/8,500 cells. Data are presented as violin plot, n=3.

C-E. Percentage of cells in anaphase (C) and prophase (D) normalized over total nuclei count, and representative images of immunostaining for aurora B kinase (green; phalloidin is red and nuclei are blue) (E) after incubating MCF7 cells for 24h with vehicle (grey), EVs from controls (blue), or EVs from cases (orange). EVs were used at a final concentration of 1 × 10^8^/8,500 cells. Data are presented as violin plot, n=3.

**Bibliography**

1.	Tavazzi L, Maggioni AP, Marchioli R, Barlera S, Franzosi MG, Latini R, et al. Effect of n-3 polyunsaturated fatty acids in patients with chronic heart failure (the GISSI-HF trial): a randomised, double-blind, placebo-controlled trial. *Lancet* 2008;**372**:1223-1230. doi: https://doi.org/10.1016/S0140-6736(08)61239-8

2.	Ameri P, Canepa M, Nicolosi GL, Marchioli R, Latini R, Tavazzi L, et al. Cancer in chronic heart failure patients in the GISSI-HF trial. *Eur J Clin Invest* 2020;**50**:e13273. doi: <https://doi.org/10.1111/eci.13273>

3.	Welsh J, Goberdhan DCI, O'Driscoll L, Buzas EI, Blenkiron C, Bussolati B, et al. Minimal information for studies of extracellular vesicles (MISEV2023): From basic to advanced approaches. *J Extracell Vesicles* 2024;**13**:e12451. doi: <https://doi.org/10.1002/jev2.12451>

4.	Käll L, Canterbury JD, Weston J, Noble WS, MacCoss MJ. Semi-supervised learning for peptide identification from shotgun proteomics datasets. *Nat Methods* 2007;**4**:923-925. doi: <https://doi.org/10.1038/nmeth1113>

5.	Vizcaíno J, Csordas A, del-Toro N, Dianes JA, Griss J, Lavidas I, et al. 2016 update of the PRIDE database and its related tools. *Nucleic Acids Res* 2016;**44**:D447-456. doi: <https://doi.org/10.1093/nar/gkv1145>

6.	Fonseka P, Pathan M, Chitti SV, Kang T, Mathivanan S. FunRich enables enrichment analysis of OMICs datasets. *Journal of Molecular Biolog* 2021;**433**:166747. doi: <https://doi.org/10.1016/j.jmb.2020.166747>

7.	Szklarczyk D, Gable AL, Nastou KC, Lyon D, Kirsch R, Pyysalo S, et al. The STRING database in 2021: customizable protein-protein networks, and functional characterization of user-uploaded gene/measurement sets. *Nucleic Acids Res* 2021;**49**:D605-612. doi: <https://doi.org/10.1093/nar/gkaa1074>

8.	Shannon P, Markiel A, Ozier O, Baliga NS, Wang JT, Ramage D, et al. Cytoscape: a software environment for integrated models of biomolecular interaction networks. *Genome Res* 2003;**13**:2498-2504. doi: <https://doi.org/10.1101/gr.1239303>

9.	Pilco-Ferreto N, Calaf GM. Influence of doxorubicin on apoptosis and oxidative stress in breast cancer cell lines. *International Journal of Oncology* 2016;**49**:753-762. doi: <https://doi.org/10.3892/ijo.2016.3558>

10.	Costes S, Daelemans D, Cho EH, Dobbin Z, Pavlakis G, Lockett S. Automatic and quantitative measurement of protein-protein colocalization in live cells. *Biophys J* 2004;**86**:3993-4003. doi: <https://doi.org/10.1529/biophysj.103.038422>
